# Body condition, age, and sex as modulators of peripheral blood immune-cell populations and lymphocyte proliferation in cats

**DOI:** 10.3389/fvets.2026.1943507

**Published:** 2026-09-09

**Authors:** Hanna Krüger, Andreas Moritz, Berit Roshani, Stephan Neumann

**Affiliations:** 1 Institute of Veterinary Medicine, Georg-August University Göttingen, Göttingen, Germany; 2 Clinic of Small Animals-Internal Medicine, Faculty of Veterinary Medicine, Justus-Liebig-University Giessen, Giessen, Germany; 3 Unit of Infection Models, German Primate Centre, Leibniz-Institute for Primate Research Göttingen, Göttingen, Germany

**Keywords:** feline obesity, flow cytometry, lymphocyte proliferation, body condition score, sex differences

## Abstract

Feline obesity is common, and in other species it has been linked to immune dysregulation; yet its immunological consequences in cats, and their modulation by age and sex, remain poorly defined. Whole blood from 78 neutered client-owned cats (37 males, 41 females; 1–16 years) was analyzed by flow cytometry using a panel characterizing T cells (including CD5^+^CD4^+^ and CD5^+^CD8^+^ subsets), B cells (CD21^+^) and natural killer (CD56^+^) cells with their activation (CD80) and proliferation (Ki67) status. Cats were grouped by body condition score into normal-weight (4–5) and overweight (6–9); lymphocyte proliferation was assessed in a subset by a carboxyfluorescein succinimidyl ester assay after concanavalin A stimulation, and serum amyloid A, triglycerides and adiponectin were measured in overweight cats. Across the cohort, overweight cats displayed higher frequencies of proliferating CD21^+^Ki67^+^ B cells than normal-weight cats, while the proliferation assay was descriptively consistent with enhanced T-cell proliferation in both sexes. Increasing age was associated with a decline in CD5^+^CD4^+^ T cells in both sexes and, in females, additionally in CD5^+^CD21^+^ cells alongside rising CD80 expression. Sex differences shifted across life, from B-cell activation in young adults to T-cell subsets in adults (higher CD5^+^CD8^+^ in males, higher Ki67^+^ fractions in females) and CD56^+^ NK cells in the oldest cats. Body-condition effects were largely age- and sex-specific and emerged only after stratification: adult overweight males showed higher proliferating CD4^+^Ki67^+^ and CD8^+^Ki67^+^ T cells, young-adult overweight females higher CD5^+^ and activated CD5^+^CD21^+^CD80^+^ cells, and aged overweight females fewer proliferating CD5^+^CD21^+^Ki67^+^ B cells. Among overweight cats, higher adiponectin was associated with fewer CD5^+^ lymphocytes, with no associations for serum amyloid A or triglycerides. Overall, feline obesity was associated with subtle changes in immune-cell composition but more pronounced effects on lymphocyte proliferative responsiveness, both strongly modulated by age and sex.

## 1 Introduction

Overweight and its associated comorbidities represent major challenges in both human and veterinary medicine (1, 2). Cats, the most commonly kept companion animals in Europe (3), are no exception. Epidemiological studies report the prevalence of feline overweight and obesity ranging from 11.5 to 63% across different countries and populations (4–7). Feline obesity is clinically relevant, as it is associated with a range of disorders—including diabetes mellitus, lower urinary tract disease, musculoskeletal disease, and dermatological conditions, as well as alterations in cardiac structure and function—that can substantially impair quality of life and reduce life expectancy (8–10).

Obesity is increasingly recognized as a systemic immunometabolic disorder characterized by chronic, low-grade inflammation rather than a process confined to adipose tissue (11). In humans and rodent models, it is accompanied by dysregulation of the adaptive immune system, including expansion of proinflammatory CD4^+^ and CD8^+^ T cells (12, 13), shifts in peripheral B-cell subsets (14), and increased lymphocyte activation and proliferation that is mirrored in the circulation (15). Adipokines such as adiponectin link metabolic status to immune function (16, 17) and, in cats, help distinguish metabolically benign from pathological obesity (18). For the cat, however, comparative data on circulating immune-cell composition and proliferative responses between normal-weight and overweight animals remain scarce (19, 20).

Immune-cell composition is itself strongly shaped by age and sex. With advancing age, hallmarks of immunosenescence emerge across species, including a decline in circulating CD4^+^ T cells (21, 22), alongside a chronic, low-grade inflammatory state termed “inflammaging” that is also described in companion animals (23, 24). Pronounced sex differences across T-, B-, and NK-cell compartments arise partly independently of gonadal hormones - for example through X-linked gene-dosage effects - and may shift over the lifespan (25–27). This argues for age- and sex-stratified rather than pooled analyses when investigating immune dysregulation in feline obesity. Against this background, the aim of the present study was to characterize the composition and proliferative activity of circulating immune-cell populations in normal-weight and overweight cats, while systematically accounting for the modulating influence of age and sex. Using flow cytometry, T-, B-, and NK-cell populations were assessed in peripheral blood together with the activation marker CD80 and the proliferation marker Ki67. This was complemented by a carboxyfluorescein succinimidyl ester (CFSE)-based proliferation assay following concanavalin A stimulation. In overweight cats, the metabolic markers serum amyloid A (SAA), triglycerides, and adiponectin were additionally measured to explore associations between metabolic status and immune-cell composition. These markers reflect systemic inflammation (SAA), lipid metabolism (triglycerides), and adipose tissue-derived immunomodulation (adiponectin), respectively.

## 2 Materials and methods

### 2.1 Animals

For this study, we analyzed EDTA whole-blood samples from 78 neutered client-owned cats (37 males, 41 females; age-range 1–16 years). Blood was collected during a routine health check-up at the Clinic for Small Animals, Veterinary Institute, Georg-August-Universität Göttingen. All cats were considered healthy based on a general clinical examination, a complete blood count, and a serum biochemistry panel within the reference ranges (Supplementary Table 1). Body condition was assessed by a single veterinarian during clinical examination using the 9-point body condition score (BCS) system (28), with cats scored 4–5 classified as normal-weight and 6–9 as overweight. For the CFSE-based proliferation assay, a subset of 12 animals was used, comprising three overweight (BCS 6–9) and three normal-weight (BCS 4–5) cats of each sex. These animals met the same inclusion and health criteria as the overall study population.

### 2.2 Sample collection

For flow cytometric analysis, blood samples were collected from the cephalic vein (Vena cephalica antebrachii) using a sterile single-use needle (20 G, 0.9 × 40 mm) and transferred into 2 ml EDTA tubes (Sarstedt, Nümbrecht, Germany). For the ELISA assays, blood was collected into 1.3 ml serum tubes (Sarstedt, Nümbrecht, Germany). Both the surface/intracellular staining for immunophenotyping and the CFSE labeling for the proliferation assay were performed immediately after sample collection.

### 2.3 Surface and intracellular staining

The markers used for the surface and intracellular staining are listed in Table 1. Staining was performed immediately after sample collection, following a previously published protocol and gating strategy (29). Briefly, the antibodies were dispensed into 5 ml tubes, to which 150 μl of EDTA whole blood was added. The biotin-conjugated CD5 antibody and Streptavidin-Brilliant Violet 421 were pre-incubated for 15 min at room temperature (RT) in the dark before being combined with the remaining surface antibodies; this mix was then incubated with the blood for a further 30 min (at RT in the dark). Subsequently 1 ml of RBC lysis/fixation solution (BioLegend, San Diego, CA, USA) was added, followed by a 15 min incubation in the dark at RT, after which the cells were centrifuged (1,200 rpm, 5 min) and the supernatant discarded. For intracellular detection of Ki67, the fixed cells were permeabilized by washing twice with intracellular staining permeabilization wash buffer (BioLegend, San Diego, CA, USA; 1,200 rpm, 5 min), discarding the supernatant after each step. The Ki67 antibody was then prepared in 50 μl of permeabilization wash buffer, added to the cell pellet, and incubated for 20 min at room temperature in the dark. Finally, the cells were washed once more with permeabilization wash buffer, centrifuged (1,200 rpm, 5 min), and resuspended in 150 μl PBS containing 5% BSA for acquisition.

**Table 1:** Staining panel.

Antibody	Clone	Label	Vendor
CD4	3-4F4	FITC	Invitrogen
CD5	f34	Streptavidin +BV421	BioLegend
CD8	fCD8	PE	Invitrogen
CD21	B-Ly4	APC	BD Bioscience
CD56	NCAM16.2	BV650	BD Bioscience
CD80	2D10	PE-Cy7	BioLegend
Ki67	B56	PerCP-Cy5.5	BD Bioscience

### 2.4 Isolation of PBMCs

Prior to CFSE staining, peripheral blood mononuclear cells (PBMCs) were isolated from 1 ml of EDTA whole blood. For this, 2 ml of 97% Pancoll human (PAN-Biotech GmbH, Aidenbach, Germany) was placed into 4.5 ml tubes, and the EDTA blood was carefully layered on top and centrifuged for 25 min at 800 × g. The separated PBMC layer was carefully transferred into a 15 ml Falcon tube containing 9 ml PBS, followed by a second centrifugation for 10 min at 1,200 × g. After the supernatant had been discarded, the cell pellet was resuspended in 2 ml PBS. For each animal, three culture wells (two with ConA, one with medium only) were prepared with 5 × 10^5^ cells each for CFSE staining.

### 2.5 CFSE staining

CFSE labeling was performed using the CFSE Cell Division Tracker Kit (BioLegend, San Diego, CA, USA) according to the manufacturer's instructions. Briefly, PBMCs were resuspended in 1 mL of CFSE working solution and incubated for 20 min at room temperature in the dark. Subsequently, 5 ml of RPMI (Roswell Park Memorial Institute) 1,640 medium supplemented with 10% fetal bovine serum (FBS) were added, and the cells were centrifuged for 10 min at 1,200 × g. The supernatant was discarded and the cell pellet resuspended in 470 μl of RPMI/10% FBS. For each cat, three wells were prepared with 150 μl of this suspension each. Concanavalin A (ConA) was dissolved in RPMI/10% FBS (4 μl ConA per 1 ml medium), and 50 μl of this solution were added to two of the three wells per cat; to the third well, 50 μl of medium were added. Cells were incubated for 6 days at 37 °C in the dark. For surface and live/dead staining (Zombie Yellow, BioLegend, San Diego, CA, USA), the two ConA wells were pooled into a 5 ml tube, while the medium-only well was transferred to a separate 5 ml tube. Staining was performed as described in Section 2.3.

### 2.6 Flow cytometry

Samples were acquired on a Sony ID7000 spectral cell analyzer (Sony Biotechnology, San Jose, CA, USA). Spectral unmixing was performed using single-stained reference controls. Flow cytometry data were analyzed using FlowJo software, version 10.10.0 (BD Biosciences, Ashland, OR, USA). Data were analyzed following a previously published gating strategy (29) (Supplementary Figure 1). After exclusion of doublets, lymphocytes were gated as singlets and separated by CD5 and CD21 expression into CD5^+^CD21^+^ (CD5^+^), CD5^+^CD21^+^ (CD21^+^), CD5^+^CD21^+^ and CD5^+^CD21^+^ populations. CD56^+^ NK cells were gated from the CD5^+^CD21^+^ fraction. CD4 and CD8 were assessed within the CD5^+^ (CD5^+^CD21^+^) population, and CD80 within the CD21^+^ and CD5^+^CD21^+^ populations, respectively; Ki67 was assessed within each respective parent population. Accordingly, all frequencies are expressed as percentages of their respective parent population.

Before each acquisition, instrument performance and optical alignment were verified using the manufacturer's quality control procedure. Spectral unmixing was performed using single-stained reference controls prepared with UltraComp eBeads Plus Compensation Beads (Invitrogen/Thermo Fisher Scientific, Waltham, MA, USA).

### 2.7 Quantification of adiponectin and SAA

Blood was collected into serum tubes, allowed to clot at room temperature for 15 min, and centrifuged at 1,600 × g for 10 min. Serum was aliquoted and stored at [−20 °C] for a maximum of 30 days until analysis. Serum concentrations of adiponectin and serum amyloid A (SAA) were determined by enzyme-linked immunosorbent assay (ELISA). Adiponectin was measured using the Cat Adiponectin ELISA Kit (MyBioSource, San Diego, CA, USA; assay range: 1.25 μg/ml−40 μg/ml) with 50 μl of serum per well, and SAA using the Feline Serum Amyloid A Detection Kit (Chondrex, Woodinville, WA, USA; assay range 31–2,000 ng/ml) with 100 μl of serum per well. Both assays were performed according to the manufacturers' instructions, and all samples were analyzed in duplicate. Absorbance was measured at 450 nm for adiponectin and at 450 nm with a reference wavelength of 630 nm for SAA, using a Sunrise microplate reader (Tecan, Männedorf, Switzerland). Analyte concentrations were calculated from the respective standard curves using Magellan software (version 7.5; Tecan, Männedorf, Switzerland). Analyte concentrations fell within the linear range of the respective standard curve.

### 2.8 Measurement of triglycerides

Serum triglyceride concentrations were measured using the Catalyst One chemistry analyzer with feline triglyceride slides (IDEXX Laboratories, Westbrook, ME, USA) according to the manufacturer's instructions, using 150 μl of serum per measurement.

### 2.9 Statistical analysis

Statistical analyses were performed using GraphPad Prism 10.1.1 (GraphPad Software, Boston, MA, USA). Distribution of the data was assessed using the Shapiro–Wilk test. As several immune-cell populations deviated from normality and showed right-skewed distributions with values close to zero, non-parametric methods were used throughout.

**Figure 1:**
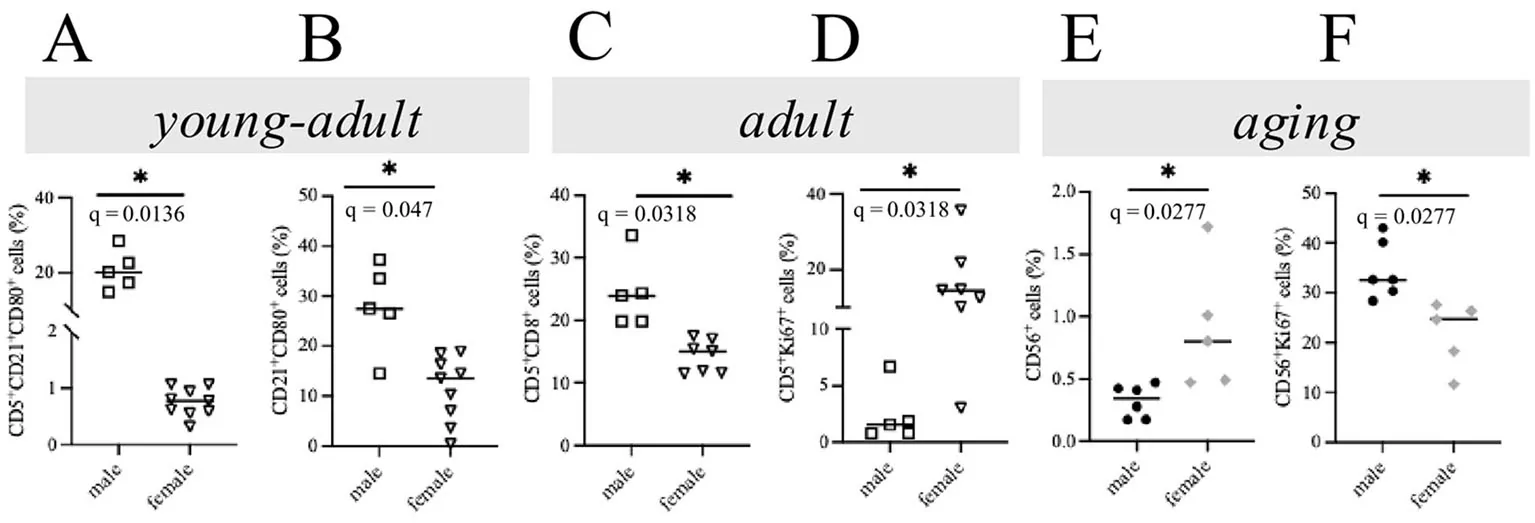
Comparison of immune-cell frequencies between male and female cats within age groups. **(A)** Frequencies of CD5^+^CD21^+^CD80^+^ cells and **(B)** CD21^+^CD80^+^ cells in male and female cats within the young-adult age group. **(C)** Frequencies of CD5^+^CD8^+^ cells and **(D)** CD5^+^Ki67^+^ cells in male and female cats within the adult age group. **(E)** Frequencies of CD56^+^ cells and **(F)** CD56^+^Ki67^+^ cells in male and female cats within the aging age group. Comparisons were performed within a single body-condition stratum per age group: normal-weight cats (BCS 4–5) for panels **(A)**–**(D)** and overweight cats (BCS 6–9) for panels **(E)** and **(F)**; open symbols represent normal-weight and filled symbols overweight animals. Median is shown. Statistical analysis was performed using the Mann–Whitney *U*-test with FDR correction; significance is based on *q*-values (*q < 0.05).

**Figure 2:**
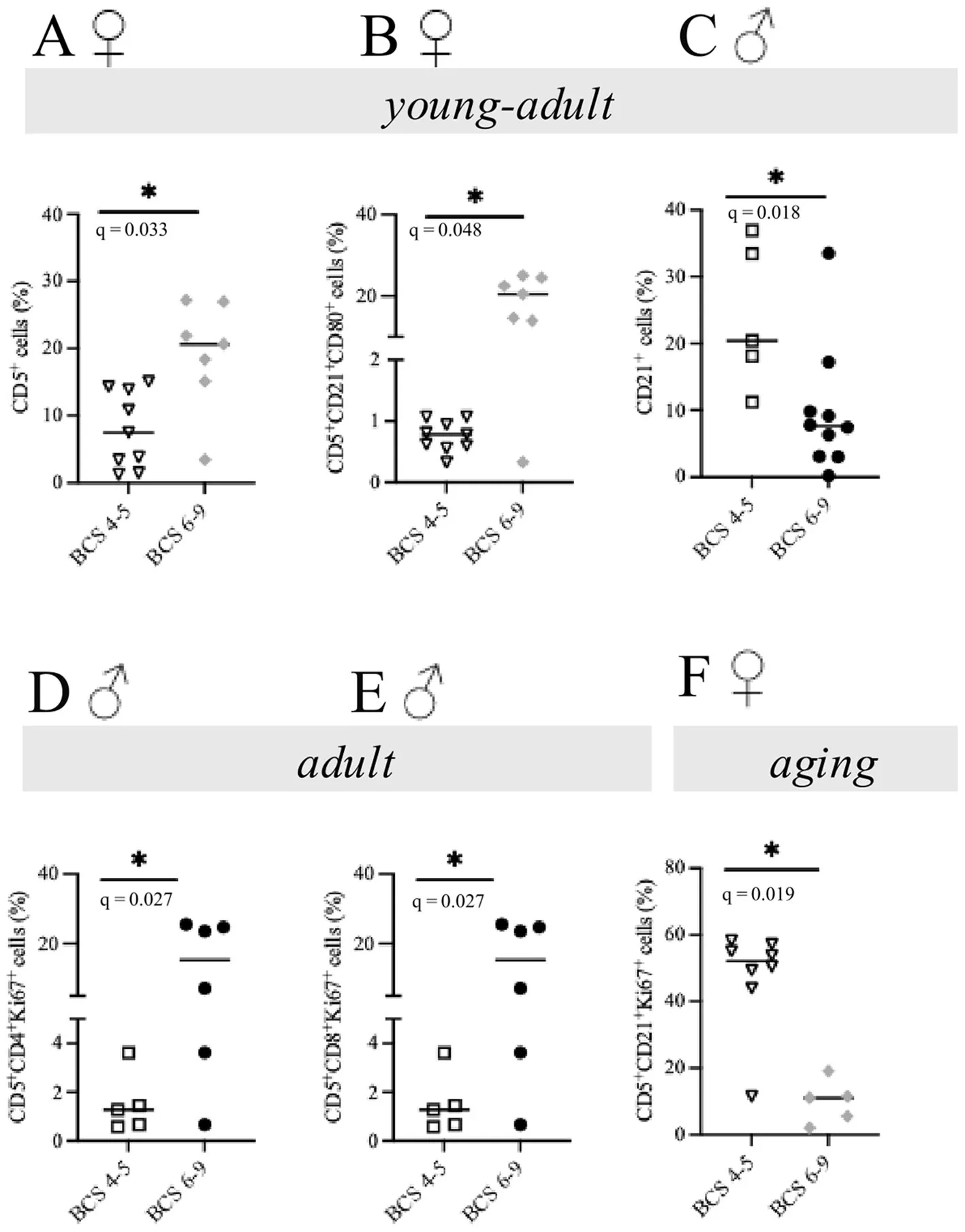
Comparison of immune-cell frequencies between normal-weight (BCS 4–5) and overweight (BCS 6–9) cats within age- and sex-defined subgroups (♀ female, ♂ male). **(A)** Frequencies of CD5^+^ cells and **(B)** CD5^+^CD21^+^CD80^+^ cells in normal-weight and overweight female cats within the young-adult age group. **(C)** Frequencies of CD21^+^ cells in normal-weight and overweight male cats within the young-adult age group. **(D)** Frequencies of CD5^+^CD4^+^Ki67^+^ cells and **(E)** CD5^+^CD8^+^Ki67^+^ cells in normal-weight and overweight male cats within the adult age group. **(F)** Frequencies of CD5^+^CD21^+^Ki67^+^ cells in normal-weight and overweight female cats within the aging age group. Open symbols represent normal-weight (BCS 4–5) and filled symbols overweight (BCS 6–9) animals. Median is shown. Statistical analysis was performed using the Mann–Whitney *U*-test with FDR correction (*q < 0.05).

Comparisons between two groups were performed using the Mann–Whitney *U*-test. To account for multiple comparisons, *p*-values were corrected using the two-stage step-up procedure of Benjamini et al. (30), controlling the false discovery rate at *Q* = 5%, with all immune-cell markers within a given comparison treated as one family. For the overall comparison between normal-weight (BCS 4–5) and overweight (BCS 6–9) cats, all immune-cell populations were tested in the pooled study population. For the analysis of sex differences within age groups, males and females were compared separately in normal-weight and in overweight cats within each age group (young-adult, adult, aging), across all markers; each of these age- and body-condition-defined comparisons was corrected independently, and only comparisons remaining significant after correction are shown (Figure 1). Body-condition comparisons within age- and sex-defined subgroups (Figure 2) were analyzed in the same manner.

Associations between age or serum adiponectin concentration and immune-cell proportions were assessed using Spearman rank correlation, with the correlation coefficient (r_s), the number of animals (*n*) and the exact *p*-value reported for each analysis. These correlation analyses were not corrected for multiple comparisons.

An *a priori* power analysis was performed using G^*^Power (version 3.1) (31). As no standardized effect-size estimates were available for obesity-associated peripheral immune-cell alterations in cats, the study was powered to detect a medium-to-large effect (Cohen's *d* = 0.65), defined *a priori* as the smallest biologically meaningful difference for the markers of interest. For the primary comparison between overweight and normal-weight cats (Mann–Whitney *U*-test, α = 0.05, power = 0.80), this yielded 39 animals per group (total *n* = 78). Subgroup comparisons were considered exploratory and were not formally powered.

## 3 Results

### 3.1 Study population and immune-cell composition

In total, we analyzed 78 cats consisting of 41 females and 37 males in the age range of 1–16 years. Animals were divided into two groups based on their body condition score (BCS) as normal-weight (BCS 4–5, 24 females, 15 males, age range 1–16 years) and over-weight cats (BCS 6–9, 17 females, 22 males, age range 2–16 years) (Table 2). For the analysis of immune cell populations, a previously published staining protocol and gating strategy was used (29) (Supplementary Figure 1). In this study, the proliferation marker Ki67 was analyzed in CD4^+^ and CD8^+^ T cells as well as in CD5^+^CD21^+^ lymphocytes. In addition, CD80 expression was assessed in CD21^+^ cells. In overweight cats (BCS 6–9), serum concentrations of serum amyloid A (SAA), triglycerides (TG), and adiponectin (APN) were additionally measured. The frequencies of immune cell populations in cats with a BCS of 4–5 are summarized in Table 3 and those for cats with a BCS of 6–9 are presented in Table 4. Median values and interquartile ranges are presented separately for male and female cats, as well as for the total study population. Considerable variability was observed across all analyzed cell populations.

**Table 2:** Demographic characteristics of the study population.

Characteristic	Normal-weight (BCS 4–5)	Overweight (BCS 6–9)
*n*	39	39
Females/males	24/15	17/22
BCS, median (range)	5 (4–5)	7 (6–9)
Age (years), median (range)	8 (1–16)	6 (1–16)

**Table 3:** Cell population analysis for felines with BCS 4–5.

Group	CD5^+^	CD5^+^CD4^+^	CD5^+^CD8^+^	CD5^+^CD21^+^	CD21^+^	CD56^+^
Total (*n* = 39)	5.39 [2.91–14.42]	43.44 [19.38–55.63]	17.50 [11.95–24.10]	0.13 [0.08–0.69]	6.58 [1.42–17.50]	0.39 [0.27–0.97]
Male (*n* = 15)	14.51 [4.43–31.94]	43.91 [17.89–55.50]	19.80 [15.95–24.10]	0.19 [0.10–0.70]	16.75 [1.61–22.20]	0.46 [0.28–0.88]
Female (*n* = 24)	4.53 [1.18–9.38]	40.86 [19.42–55.46]	16.20 [11.90–22.73]	0.11 [0.08–0.69]	4.82 [1.42–11.75]	0.39 [0.25–0.89]
*q*-value	0.0256^*^	0.7846	0.5390	0.5390	0.5390	0.7093

Data are median [interquartile range, 25th−75th percentile]. Male vs. female were compared using the Mann–Whitney U-test with Benjamini–Krieger–Yekutieli FDR correction across all markers; ^*^q < 0.05.

**Table 4:** Cell population analysis for felines with BCS 6–9.

Group	CD5^+^	CD5^+^CD4^+^	CD5^+^CD8^+^	CD5^+^CD21^+^	CD21^+^	CD56^+^
Total (*n* = 39)	8.51 [3.07–18.29]	43.28 [30.58–56.18]	18.30 [12.70–23.65]	0.23 [0.15–0.51]	3.16 [1.48–9.29]	0.41 [0.20–0.69]
Male (*n* = 22)	6.78 [1.22–16.03]	41.42 [31.89–59.77]	17.45 [12.60–22.60]	0.19 [0.05–0.35]	4.12 [1.88–8.80]	0.31 [0.18–0.49]
Female (*n* = 17)	15.10 [3.46–20.53]	43.28 [30.38–54.16]	19.50 [12.90–28.00]	0.49 [0.18–0.75]	2.68 [1.47–9.44]	0.49 [0.29–0.83]
*q*-value	0.2724	0.6512	0.6512	0.2354	0.6512	0.2724

Data are median [interquartile range, 25th−75th percentile]. Male vs. female were compared using the Mann–Whitney U-test with Benjamini–Krieger–Yekutieli FDR correction across all markers; no comparison reached significance.

Male cats with a BCS of 4–5 showed a significantly higher proportion of CD5^+^ cells compared to females.

### 3.2 Comparison of immune-cell populations and proliferation between body condition groups

**Figure 3:**
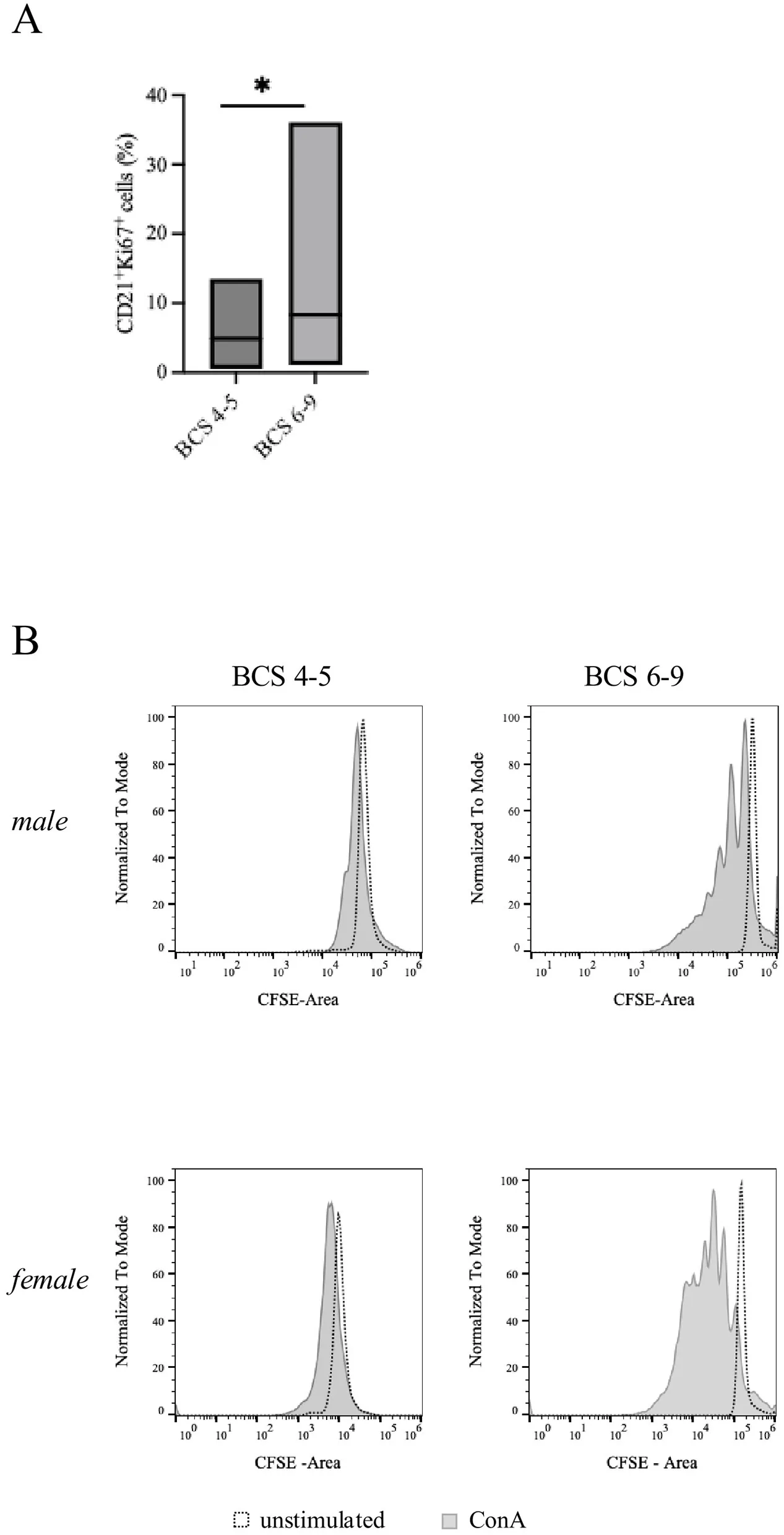
Feline lymphocyte proliferation in normal-weight and overweight cats. **(A)** Frequencies of CD21^+^Ki67^+^ cells in normal-weight (BCS 4–5, *n* = 39) and overweight (BCS 6–9, *n* = 39) cats across the entire study population. Median is shown. Statistical analysis was performed using the Mann–Whitney *U*-test with false-discovery-rate correction (two-stage step-up procedure of Benjamini, Krieger and Yekutieli, *Q* = 5%); *q < 0.05. **(B)** Representative CFSE histograms demonstrating CD5^+^CD8^+^ T cell proliferation for each sex (*males: upper panel, females: lower panel*) and group (*normal weight: left panel, overweight: right panel*) following ConA stimulation. Dotted line, unstimulated; gray, ConA-stimulated.

Next, we compared cats with an ideal body condition (BCS 4–5) and overweight cats (BCS 6–9) regarding differences in immune cell populations and proliferation using a CFSE proliferation assay. Across the entire study population, we observed a significant difference only for CD21^+^Ki67^+^ cells as cats with BCS 6–9 showed a higher proportion of those cells compared to normal weight cats (Figure 3A; *q* = 0.041).

We also performed a CFSE-based proliferation assay to compare T- and B-cell proliferative responses to ConA stimulation between normal-weight and overweight cats, using three animals of each sex per BCS group (age range: 2–16 years). Figure 3B shows representative CFSE histograms of CD5^+^CD8^+^ T cells for each sex and group. In both sexes, overweight cats displayed a more pronounced CFSE dye dilution than normal-weight cats, indicative of a higher number of cell divisions following ConA stimulation. As this assay was performed in a limited number of animals and served a descriptive, illustrative purpose, no statistical analysis was conducted; the histograms are shown as representative examples.

### 3.3 Age- and sex-related effects on immune-cell populations

**Figure 4:**
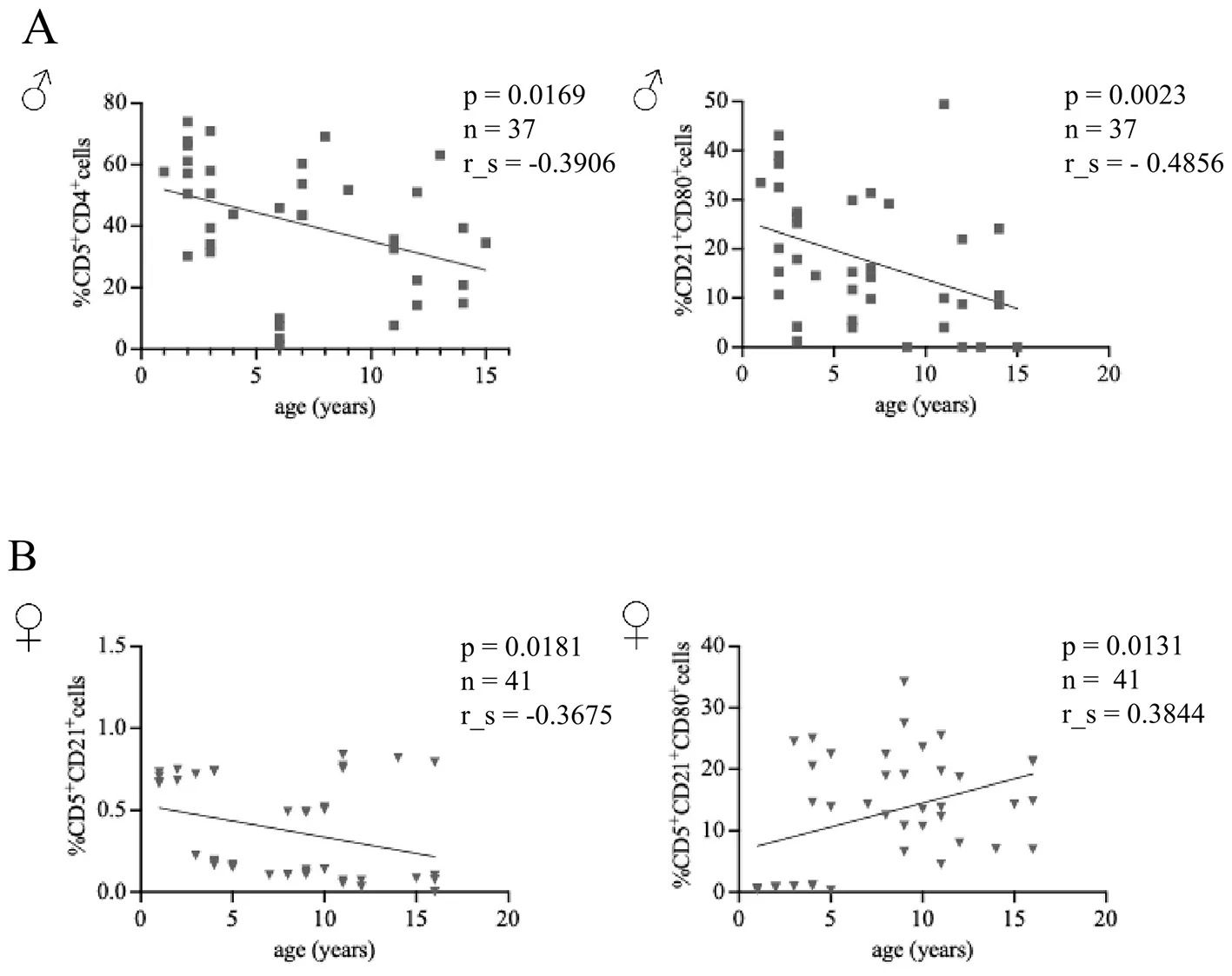
Correlation between immune-cell frequencies and age in male and female cats. Spearman rank correlation between the frequency of the respective immune-cell population and age (years) in male and female cats. **(A)** Male CD5^+^CD4^+^, male CD21^+^CD80^+^, **(B)** female CD5^+^CD21^+^, female CD5^+^CD21^+^CD80^+^. The correlation coefficient (r_s), number of animals (*n*) and exact *p*-value are given in each panel.

In general, increasing age in male cats correlated with a significant decrease in the proportion of CD5^+^CD4^+^ T lymphocytes (*p* = 0.017) and CD21^+^CD80^+^ B cells (*p* = 0.002) (Figure 4A). These correlations were of weak and moderate strength, respectively.

In female cats, aging was significantly negatively correlated with CD5^+^CD4^+^ T lymphocytes (r_s = −0.38, *p* = 0.013, *n* = 41; *data not shown*). Frequencies of CD5^+^CD21^+^ cells significantly dropped with increasing age, while their expression of CD80 significantly increased (Figure 4B).

Based on these age-related correlations, cats were assigned to three age groups—young-adult (1–4 years), adult (5–9 years) and aging (>10 years). Within each age group, males and females were compared separately in normal-weight and overweight cats across all immune-cell markers; only significant comparisons are reported below (Figure 1).

Among young-adult, normal-weight cats, males showed significantly higher proportions of CD5^+^CD21^+^CD80^+^ (Figure 1A; *q* = 0.014) and CD21^+^CD80^+^ cells (Figure 1B; *q* = 0.048) than females. Among adult, normal-weight cats, CD5^+^CD8^+^ cells were more frequent in males (Figure 1C; *q* = 0.032), whereas Ki67^+^ cells within the total CD5^+^ compartment were more frequent in females (Figure 1D; *q* = 0.032). Among aging, overweight cats, males showed significantly lower proportions of CD56^+^ NK cells (Figure 1E; *q* = 0.027) and higher proportions of CD56^+^Ki67^+^ cells (Figure 1F; *q* = 0.027) than females.

Comparative analysis between cats with ideal body condition (BCS 4–5) and overweight cats (BCS 6–9) revealed that in young-adult female cats, overweight animals showed significantly higher proportions of CD5^+^ (Figure 2A; *q* = 0.033) and CD5^+^CD21^+^CD80^+^ cells (Figure 2B; *q* = 0.048). In young-adult, normal weight males CD21^+^ B cells were significantly more abundant compared to overweight males (Figure 2C; *q* = 0.018). Adult, overweight males exhibited significantly higher proportions of CD4^+^Ki67^+^ (Figure 2D; 0.027) and CD8^+^Ki67^+^ T cells (Figure 2E; *q* = 0.027).

In females aged >10 years, CD5^+^CD21^+^Ki67^+^ cells were significantly more abundant in cats with BCS 4–5 compared to overweight individuals (Figure 2F; *q* = 0.020).

### 3.4 Associations between metabolic markers and immune-cell populations in overweight cats

To further investigate potential relationships between metabolic status and immune cell composition, association analyses were performed within the overweight cat population using the metabolic parameters Serum Amyloid A (SAA), Adiponectin (APN) and triglycerides (TG). These parameters were assessed in overweight cats specifically, as they reflect the metabolic and inflammatory status associated with excess body condition and help distinguish metabolically benign from pathological obesity (18), whereas in normal-weight cats they would be expected to show little variation.

**Figure 5:**
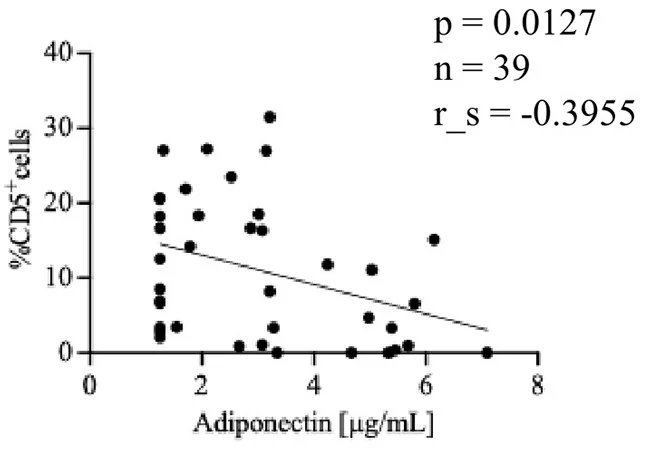
Correlation between CD5^+^ and adiponectin level. Spearman rank correlation between CD5^+^ cell frequency and serum adiponectin in overweight cats (BCS 6–9). The correlation coefficient (r_s), number of animals (*n*) and exact *p*-value are given in the panel.

In the overweight cat group, increasing APN levels were significantly associated with a decrease in the proportion of CD5^+^ lymphocytes, indicating a negative linear relationship (Figure 5; *p* = 0.013). As expected, BCS was negatively correlated with adiponectin within the overweight group (r_s = −0.48, *p* = 0.002, *n* = 39), consistent with the well-documented decline of circulating adiponectin with increasing adiposity. No significant correlations were observed between BCS and SAA or TG.

## 4 Discussion

The present study demonstrates that obesity-associated immune alterations in cats are subtle and strongly modulated by age and sex, underlining the importance of stratified analyses when investigating immune dysregulation in feline obesity.

Across the entire cohort, overweight cats showed a significantly higher proportion of CD21^+^Ki67^+^ cells compared to normal-weight animals—the sole significant difference identified between BCS groups. While obesity-associated alterations in B-cell subset composition have been described in human peripheral blood (15, 32) comparable data from peripheral blood of companion animals remain scarce, and changes in proliferating CD21^+^ B cells in feline obesity have, to our knowledge, not previously been reported.

The subsequent CFSE-based assay, although limited to few animals per group and assessed descriptively, yielded histograms consistent with enhanced T-cell proliferation in overweight cats of both sexes. While lymphocyte proliferation in cats has been assessed using colorimetric and radioactive methods (19, 20) and CFSE-based assays have been applied in the feline context (33), data on lymphocyte proliferative responses in cats remain limited overall. To the authors' knowledge, this is the first use of a CFSE dye-dilution assay to compare proliferative responses between normal-weight and overweight cats and warrants confirmation in larger cohorts.

Age- and sex-related effects substantially influenced immune-cell composition. In both sexes, increasing age was associated with a significant decline in CD5^+^CD4^+^ T lymphocytes, consistent with immunosenescence, including age-associated reductions in circulating CD4^+^ T cells (21, 22), and with the broader concept of inflammaging described across species including companion animals (24, 34, 35). In female cats only the age-associated decline in CD5^+^CD21^+^ lymphocytes was accompanied by increased CD80 expression. The decline is broadly in line with sex-divergent dynamics of CD21^low^ B cells in humans, where CD21low proportions decreased with age in females but increased in males (36)—although CD21low B cells are a distinct subset rather than the CD21-positive population analyzed here, so this comparison should be interpreted with caution. While increased CD80 expression on activated and age-associated B-cell subsets is described in mice and humans (37, 38), the concurrent CD80 increase within a contracting CD5^+^CD21^+^ population, restricted to females, has to our knowledge, not been reported previously.

CD80-expressing B cells also differed between sexes in the youngest cohort, but in the opposite direction: CD5^+^CD21^+^CD80^+^ and CD21^+^CD80^+^ cells were more frequent in young-adult males. This is notable given that B-cell activation is hormonally modulated, with estrogens enhancing B-cell activation and survival (39) and progesterone suppressing costimulatory molecules such as CD80 (40). As all cats were neutered, a gonadal hormone-driven mechanism appears unlikely to fully account for this difference. Sex-based differences in immune function can also arise independently of circulating sex hormones—through X-linked gene dosage effects, escape from X-chromosome inactivation, or organizational hormonal effects established earlier in life (26)—although these do not readily explain the male-biased direction observed here. Taken together, the sex differences across the three age cohorts did not affect a single consistent population but involved distinct compartments at different ages: B-cell activation in young adults, T-cell subsets in adults (CD5^+^CD8^+^ higher in males, Ki67^+^ within CD5^+^ higher in females), and NK cells in the oldest group. Rather than a static sexual dimorphism, this suggests that the compartments distinguishing the sexes change across the lifespan, as described in a murine model of age-dependent, sex-specific reorganization of circulating B-, T- and NK-cell compartments (27). As our study was cross-sectional, these observations describe cohort-level patterns rather than within-individual trajectories.

In the oldest cohort, total CD56^+^ cells were more abundant in females, whereas the proliferating CD56^+^Ki67^+^ fraction was higher in males. A sex-biased NK phenotype persisting independently of gonadal hormones has been described across species, with males showing a more proinflammatory, less responsive profile and females more robust effector function, pointing to sex-chromosomal rather than sex-steroid contributions (41, 42). As these reports concern NK-cell numbers and effector function rather than Ki67-based proliferation, our findings add a proliferation-related facet rather than directly corresponding to them. Notably, the female-biased CD56^+^ numbers run counter to the male-biased NK numbers reported in humans (43) and mice (41), suggesting the sex–NK relationship may differ in cats. Stratifying each age cohort by body condition revealed further effects not apparent in the pooled comparison. In young-adult females, overweight animals showed higher proportions of CD5^+^ T cells—consistent with reports that peripheral T-cell populations expand in obesity, including in young adult humans aged 20–35 years (44, 45) and of activated CD5^+^CD21^+^CD80^+^ B cells. We note, however, that human and feline age categories are not directly equivalent, and this comparison is intended as an illustrative parallel rather than an exact age correspondence. The latter is harder to contextualize, as comparable data for this subset are lacking; CD80 is a recognized activation marker (46) and is upregulated on adipose tissue macrophages and dendritic cells in obesity (47, 48), so it can only tentatively suggest obesity-associated B-cell activation. In overweight young-adult males, by contrast, CD21^+^ B cells were less frequent—a shift reported in obesity (49) that may reflect reduced B-cell numbers or a transition toward the CD21low phenotype expanding in obesity (50).

In the adult cohort, overweight males showed higher proportions of proliferating CD5^+^CD4^+^Ki67^+^ and CD5^+^CD8^+^Ki67^+^ T cells than normal weight males. T cells play a central role in obesity-associated chronic low-grade inflammation across mice and humans, with proinflammatory CD4^+^ and CD8^+^ populations expanding in the obese state (11) and adipose and peripheral blood T-cell profiles correlate in humans (51) suggesting the increased peripheral proliferation observed here may be a circulating correlate of obesity-associated immune activation. This literature, however, describes T-cell accumulation and polarization rather than proliferation (11, 51); even deep phenotyping of circulating T cells in obesity has focused on functional subsets such as Th1, Th17 and regulatory T cells (52).

A contrasting pattern emerged in the oldest cohort, where overweight females showed lower proportions of proliferating CD5^+^CD21^+^Ki67^+^ B cells than normal-weight females. Given the age-associated decline of CD5^+^CD21^+^ lymphocytes seen in female cats here, excess body condition may act in the same direction as aging within this compartment. This is consistent with reports that obesity accelerates age-related B-cell defects, enriching the pool in dysfunctional, senescent-like subsets (53, 54); as those studies assessed B-cell function and senescence markers rather than Ki67, our finding is compatible with, but not direct evidence of, accelerated B-cell senescence. Most studies underpinning these concepts examined immune cells within adipose tissue, whereas we analyzed circulating lymphocytes. Obesity is nonetheless increasingly recognized as a systemic immunometabolic disorder, and altered circulating immune-cell populations have been reported in obese individuals (55, 56). The responses seen here may therefore represent systemic manifestations of obesity-associated immune dysregulation rather than adipose tissue-specific effects, though whether they reflect comparable processes within feline adipose tissue could not be addressed. Notably, the differences detected by CFSE appeared more pronounced than differences in cell frequencies, suggesting that obesity may primarily affect lymphocyte responsiveness rather than composition. Finally, within the overweight cohort, only adiponectin showed a significant association with immune-cell populations. Increasing adiponectin concentrations were associated with a lower proportion of CD5^+^ lymphocytes, consistent with the immunomodulatory, anti-inflammatory role attributed to adiponectin, including its capacity to restrain T-cell activation and proliferation (16, 17). Given its established role as a biomarker of metabolic status (57) that, in cats, helps distinguish metabolically benign from pathological obesity (18), this association may reflect an indirect, metabolic-status-driven relationship rather than a direct effect on these cells. No associations were observed for SAA or TG. The absence of an SAA association is consistent with the clinically healthy status of our cohort: SAA is the major feline acute-phase protein and rises primarily during acute inflammation or tissue damage, whereas uncomplicated obesity elicits little or no SAA response. In the framework of Okada et al. (18), elevated SAA characterizes “obesity disease,” the pathological, inflammatory form, whereas overweight cats without an inflammatory reaction are classified as “simple obesity,” (18) analogous to metabolically healthy obesity in humans (58). As our cats were clinically healthy and non-inflamed, they correspond most closely to this latter category, in which an SAA signal would not be expected — arguing against a generalized acute-phase response rather than against a link between metabolic status and immune-cell composition *per se*.

Several limitations should be considered. The study was cross-sectional and based on cell proportions in peripheral blood, precluding causal inference and conclusions about adipose tissue-resident populations. The age groups were defined *post hoc*, informed by the observed correlations rather than by pre-specified cut-offs, and should be regarded as exploratory. The stratified subgroup analyses rested on small numbers; with such samples, the rank-based Mann–Whitney *U*-test resolves only a limited number of discrete *p*-values, constraining power and the precision of the corresponding *q*-values. Accordingly, non-significant subgroup comparisons should not be interpreted as evidence for the absence of an effect. The correlation analyses were not corrected for multiple comparisons and are likewise exploratory. Finally, proliferation was assessed by Ki67 and CFSE without dedicated functional or senescence markers; future studies incorporating functional read-outs—such as cytokine profiling (e.g. IFN-γ, TNF-α, IL-10) or characterization of regulatory T-cell and memory subsets—would help clarify whether the proliferating cells identified here are also functionally activated. Within these constraints, our data indicate that feline obesity is associated with only subtle changes in circulating immune-cell composition but more pronounced effects on lymphocyte proliferative responsiveness, both strongly shaped by age and sex. These observations are best regarded as hypothesis-generating and warrant confirmation in larger, ideally longitudinal cohorts incorporating paired adipose tissue sampling and functional read-outs.

## 5 Conclusion

Feline obesity is associated with only subtle alterations in the composition of circulating immune cells but with more pronounced effects on lymphocyte proliferative responsiveness, both strongly modulated by age and sex. These findings highlight the need for age- and sex-stratified analyses in feline immunometabolic research, as pooled comparisons may obscure biologically relevant, subgroup-specific effects. Given the cross-sectional design and the restriction to peripheral blood, these observations should be regarded as hypothesis-generating and provide a basis for future longitudinal studies that combine peripheral blood with adipose tissue sampling and functional read-outs.

## Data Availability

The raw data supporting the conclusions of this article will be made available by the authors, without undue reservation.
