# Supplementary material for: Body condition, age, and sex as modulators of peripheral blood immune-cell populations and lymphocyte proliferation in cats
Source: Front Vet Sci. 2026 Sep 9;13:1943507. doi: 10.3389/fvets.2026.1943507 (PMC13599127; doi:10.3389/fvets.2026.1943507)
Supplement: Supplementary file 1 [file Table_1.DOCX]

| **Parameter** | **Unit** |
| --- | --- |
| Glucose | Mg/dl |
| Fructosamine | µmol/l |
| Total Bile Acids | µmol/l |
| Glutamate dehydrogenase (GLDH) | U/l |
| Alanine aminotransferase (ALT) | U/l |
| Aspartate aminotransferase (AST) | U/l |
| Total bilirubin (TBIL) | mg/dl |
| Alkaline phosphatase (ALP) | U/l |
| Total protein (TP) | g/dl |
| Albumin | g/dl |
| Creatinine | mg/dl |
| Urea | mg/dl |
| Calcium | mmol/l |
| Phosphorous | mmol/l |
| Total cholesterol | mg/dl |
| Lipase | U/l |
| Creatine kinase | U/l |
| Potassium | mmol/l |
| Sodium | mmol/l |
| Globulin | g/dl |

**Supplementary Table 1.** Serum biochemistry panel assessed in the study population. The listed parameters were measured to confirm the clinically healthy status of all cats included in the study.
